# Tropical Infectious Diseases: Still Here, Still Raging, Still Killing

**DOI:** 10.4269/ajtmh.21-1026

**Published:** 2021-10-25

**Authors:** Peter J. Hotez

**Affiliations:** ^1^ Departments of Pediatrics and Molecular Virology and Microbiology, National School of Tropical Medicine, Baylor College of Medicine, Houston, Texas;; ^2^ Department of Biology, Baylor University, Waco, Texas;; ^3^ Hagler Institute of Advanced Study and Scowcroft Institute of International Affairs, Texas A&M University, College Station, Texas;; ^4^ James A. Baker III Institute of Public Policy, Rice University, Houston, Texas

In our time of COVID-19, practically every public health agency—from local and state health departments, to the U.S. CDC, to the WHO—has concentrated its efforts on slowing SARS-2 coronavirus transmission. This occurred initially through nonpharmaceutical interventions and then, in the second year of the pandemic, through administering vaccinations. Despite these efforts, the consequences of the COVID-19 pandemic of 2019–2021 have been devastating. The most recent estimates from the Institute of Health Metrics and Evaluation at the University of Washington indicate that up to 6.5 million people will have lost their lives from COVID-19 by the end of 2021.^1^

Tragically, the deaths and disability-adjusted life years (DALYs) lost over this period will extend beyond the direct effects of SARS-2 coronavirus. For instance, in the United States and globally, the ensuing social disruptions slowed or even halted childhood vaccination programs.^2^^,^^3^ Although childhood vaccinations are rebounding as waves of the COVID-19 epidemic pass, one worry is that all of the antivaccine aggression now directed at COVID-19 vaccines may spill over to other programs. In such a case, we might not achieve pre-pandemic immunization levels for many months or even years; we might experience resurgence of measles and other vaccine-preventable infectious diseases.^4^

Another concern is the diversion of global health programs toward COVID-19 at the expense of tropical infectious diseases such as the neglected tropical diseases (NTDs) and malaria. Since 2000, with the start of Millennium Development Goals programs, there have been enormous strides made in disease burden reductions for these conditions. For the NTDs, donor support from the governments of the United States and United Kingdom and operational research from the Bill & Melinda Gates Foundation, together with an ecosystem of nongovernmental development organizations (NGDOs), health ministries, and the WHO, have contributed to enormous reductions in the prevalence of NTDs through mass drug administration and preventive treatments.^5^ This is also true for global infection control programs supported by the Global Fund to Fight AIDS, Tuberculosis and Malaria; the U.S. President’s Emergency Plan for AIDS Relief and Malaria Initiative; as well as efforts to unify these programs under the banner of universal health coverage.^6^

Two new papers in the *American Journal of Tropical Medicine and Hygiene* (*AJTMH*) examine the consequences of the COVID-19 pandemic and its adverse impact on global efforts to control tropical infectious diseases in Africa.^7^^,^^8^

In Kabore et al., an NTD consortium of U.S. government contractors and NGDOs, led by FHI360, examined the impact of the pandemic on programs of NTD control in 11 West African countries.^7^ In spring 2020, the WHO issued guidelines to postpone or delay mass treatment campaigns, only to reissue guidance in the summer for cautiously restarting these programs when feasible.^7^ In response, the NTD consortium created a tool or matrix to monitor situation reports on COVID-19 and shifting epidemiologic patterns, while modifying NTD programs to restart them safely. There was emphasis on adjusting mass drug administration approaches to minimize COVID-19 transmission. They included extra resources for programmatic staff to maintain social distancing during drug administration, often going door-to-door rather than conducting large public gatherings, and ensuring an adequate supply of personal protective equipment.^7^ Still another element included efforts to combat healthcare disinformation.

In the second paper, Bell and Hansen used disease burden calculations to determine that the DALYs lost from COVID-19 in Sub-Saharan Africa (north of South Africa and Lesotho) for the year 2020 represented only a tiny fraction (2–4%) of the DALYs from the “big three” diseases—AIDS, malaria, and tuberculosis.^8^ However, by including South Africa and Lesotho, where COVID-19 transmission was more widespread, that fraction was roughly an order of magnitude higher. Only among older populations (above 65–69 years, including South Africa and Lesotho) did the deaths from COVID-19 exceed those from HIV/AIDS, whereas malaria deaths were below those from COVID-19 only among those above 70–74 years of age.^8^

As the delta variant sweeps through sub-Saharan Africa in 2021 and into 2022, these ratios may change. If we do not find a way to immunize the African continent in the coming months, the deaths and DALYs lost from COVID-19 may indeed begin resembling those from the big three diseases and NTDs. Most global health experts feel that because of the delta variant, any advantages that Africa has had previously in staving off the worst effects of COVID-19 could soon dissipate. Therefore, there is urgency to quickly scale up production of COVID-19 vaccine doses.^9^

These *AJTMH* papers also remind the global health community of how the health effects of COVID-19 go far beyond the impact of the actual virus. In the United States, as intensive care units became overwhelmed with COVID-19 patients, some Americans with other life-threatening conditions such as myocardial infarctions or stroke were denied healthcare access. We have already seen the impact of COVID-19 on childhood vaccination programs, as highlighted earlier. It is clear, however, that we simply cannot allow this to happen with the big three diseases and NTDs. Malaria, tuberculosis, and the NTDs are ancient afflictions that have decimated the health and well-being of African populations for centuries. HIV/AIDS is of course much newer, but it is only now that mass antiretroviral treatments are beginning to have a substantial impact.

We must simply find a way to implement COVID-19 prevention measures while continuing to treat and prevent tropical infectious diseases. There is no choice. This point must become a top priority for international organizations. Therefore, finding ways to co-link COVID-19 and tropical infectious disease prevention in Africa should be a high priority for the 2022 World Health Assembly in May 2022.
